# Association between statin use and the risk for idiopathic pulmonary fibrosis and its prognosis: a nationwide, population-based study

**DOI:** 10.1038/s41598-024-58417-9

**Published:** 2024-04-02

**Authors:** Jimyung Park, Chang-Hoon Lee, Kyungdo Han, Sun Mi Choi

**Affiliations:** 1 Division of Pulmonary and Critical Care Medicine, Department of Internal Medicine, Seoul National University Hospital, Seoul National University College of Medicine, 101, Daehak-ro, Jongno-gu, Seoul, 03080 South Korea; 2 Department of Statistics and Actuarial Science, Soongsil University, Seoul, South Korea

**Keywords:** Case–control study, Idiopathic pulmonary fibrosis, Observational study, Pharmacoepidemiology, Statin, Respiratory tract diseases, Epidemiology

## Abstract

Given the pleiotropic effects of statins beyond their lipid-lowering effects, there have been attempts to evaluate the role of statin therapy in IPF, but they have shown inconclusive results. Data from the National Health Insurance Service (NHIS) database of South Korea were used to investigate the effects of statin therapy on IPF. The IPF cohort consisted of a total of 10,568 patients who were newly diagnosed with IPF between 2010 and 2017. These patients were then matched in a 1:3 ratio to 31,704 subjects from a control cohort without IPF, with matching based on age and sex. A case–control study was performed to evaluate the association between statin use and the risk for IPF, and the multivariable analysis revealed that statin use was associated with a lower risk for IPF (adjusted OR 0.847, 95% CI 0.800–0.898). Using the IPF cohort, we also evaluated whether statin use at the time of diagnosis was associated with future clinical outcomes. The statin use at the time of IPF diagnosis was associated with improved overall survival (adjusted HR 0.779, 95% CI 0.709–0.856). Further prospective studies are needed to clarify the role of statin therapy in IPF.

## Introduction

Idiopathic pulmonary fibrosis (IPF) is a prototype of progressive fibrotic lung disease. Progressive fibrosis is mainly driven by repetitive microinjuries to the alveolar epithelium, leading to aberrant repair process^1^. Although the development of anti-fibrotic drugs has slowed the decline in lung function, there is still an unmet need to improve the prognosis of patients with IPF^2^.

While aberrant epithelial-mesenchymal crosstalk is regarded to be the key pathogenetic factor in IPF, inflammation is also considered to play a role^3^. Currently approved anti-fibrotic drugs for IPF, such as pirfenidone and nintedanib, have anti-inflammatory effects in addition to their well-known anti-fibrotic effects^4,5^. Although aggressive immunosuppressive therapy has been shown to be potentially harmful in IPF^6^, an adequate level of control of inflammatory responses may be helpful^3^.

Statins are widely used to treat dyslipidemia by inhibiting 3-hydroxy-3-methylglutaryl coenzyme A (HMG-CoA) reductase. In addition to lipid-lowering effects, statins have anti-inflammatory and anti-oxidative effects^7^. In this regard, the role of statins in IPF has been evaluated in a few studies. Some studies have reported beneficial effects of statins in delaying disease progression and reducing mortality^8–11^, while others have not^12^. A recent meta-analysis also failed to provide conclusive evidence supporting the beneficial effects of statins in IPF^13^. Furthermore, most available studies have focused on the effects of statins in patients already diagnosed with IPF. Data regarding whether the use of statins could alter the risk for IPF in the general population or subjects at risk for IPF are lacking, including those with interstitial lung abnormality (ILA).

As such, we aimed to investigate the role of statin therapy in IPF and designed two distinct types of studies for this purpose. First, we conducted a case–control study to evaluate the association between statin use and the risk for IPF by comparing patients with IPF and control subjects without IPF. Second, we conducted a retrospective cohort study focusing only on the cohort of patients with IPF to evaluate whether clinical outcomes differed between statin users and nonusers among patients with IPF. A nationwide, population-based cohort data from the National Health Insurance Service (NHIS) database of South Korea was used for this study.

## Results

### Characteristics of the study population

**Figure 1:**
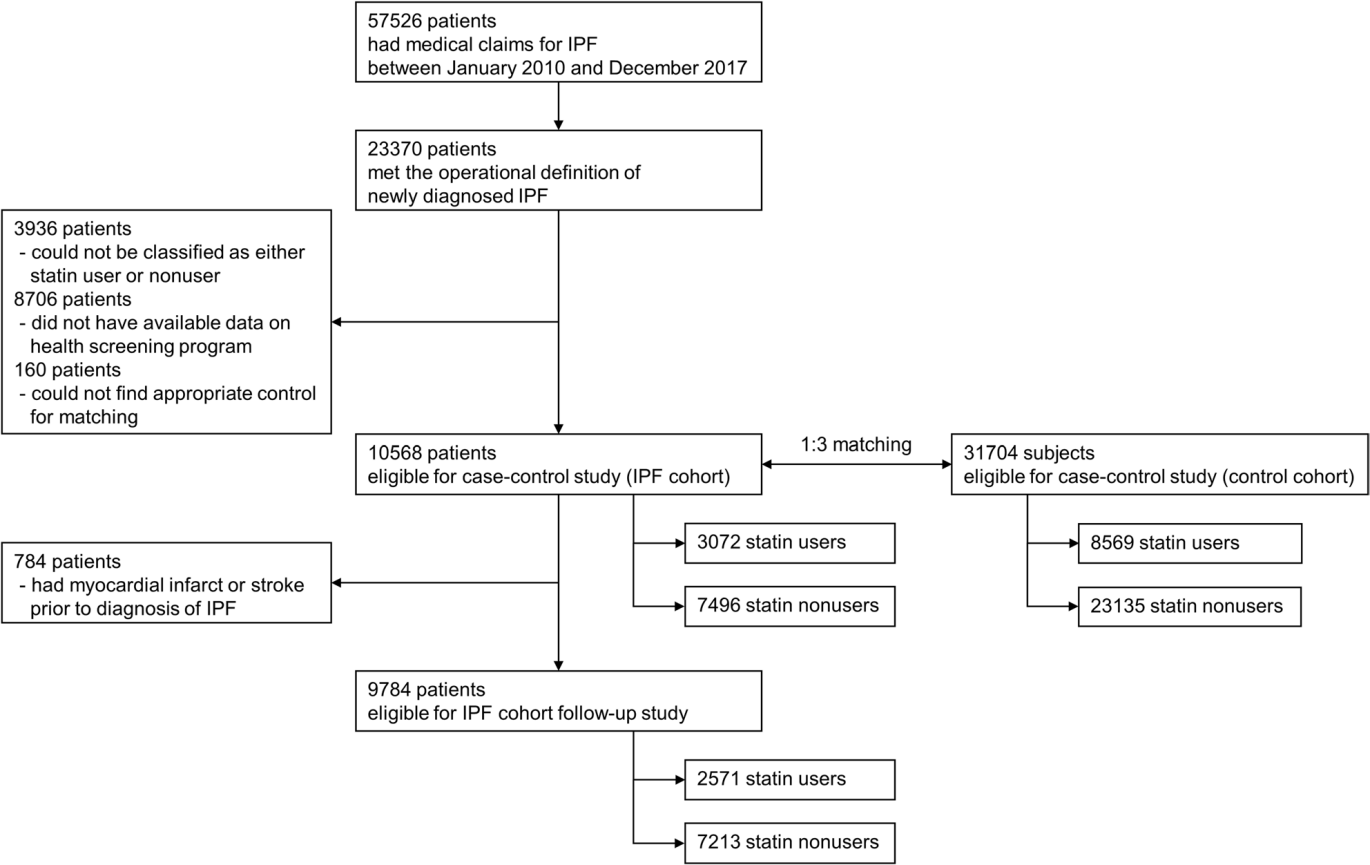
Flowchart illustrating selection of the study population.

A search of the NHIS database identified 23,370 patients newly diagnosed with IPF during the study period. After excluding patients in whom the operational definition of statin use could not be applied (i.e., only transient use) and those who did not undergo health screening programs within 2 years before the IPF diagnosis, 10,568 patients were ultimately included in the IPF cohort. For this IPF cohort, a control cohort comprising 31,704 subjects without IPF were matched at a ratio of 1:3 according to age and sex (Fig. 1). The mean age of the patients was 65 years, and 69% were male. Comorbidities related to cardiovascular diseases were more common in the IPF cohort, including diabetes and ischemic heart disease (Table 1).

**Table 1:** Baseline characteristics of patients with IPF and the matched population without IPF.

Variables	IPF (N = 10,568)	Non-IPF (N = 31,704)	*P* value
Age (yr)	65 ± 12	65 ± 12	1.000
Male sex	7269 (68.8)	21807 (68.8)	1.000
Body mass index (kg/m^2^)	23.6 ± 3.2	23.9 ± 3.1	< 0.001
Smoking status			
Never smoker	5379 (50.9)	17,855 (56.3)	< 0.001
Former smoker	2766 (26.2)	7870 (24.8)	
Current smoker	2423 (22.9)	5979 (18.9)	
Alcohol drinking			
No drinking	6806 (64.4)	19,147 (60.4)	< 0.001
Light drinking	2165 (20.5)	7425 (23.4)	
Moderate drinking	898 (8.5)	2870 (9.1)	
Heavy drinking	699 (6.6)	2262 (7.1)	
Low income^a^	2375 (22.5)	6785 (21.4)	0.021
Regular exercise^b^	2074 (19.6)	7213 (22.8)	< 0.001
Urban residency	4464 (42.2)	13,456 (42.4)	0.716
Comorbidity			
Hypertension	5309 (50.2)	16,338 (51.5)	0.021
Diabetes mellitus	2526 (23.9)	5864 (18.5)	< 0.001
Ischemic heart disease	2486 (23.5)	3786 (11.9)	< 0.001
Chronic kidney disease	1223 (11.6)	3140 (9.9)	< 0.001
Stroke	793 (7.5)	1728 (5.5)	< 0.001
Laboratory results			
Total cholesterol	185 ± 38	189 ± 38	< 0.001
LDL cholesterol	108 ± 34	110 ± 34	< 0.001
HDL cholesterol	52 ± 17	53 ± 17	< 0.001
Fasting glucose	105 ± 29	103 ± 27	< 0.001

Data are presented as mean ± standard deviation and number (%) as indicated. Patients with IPF were 1:3 matched with subjects without IPF according to age and sex.

*LDL* low-density lipoprotein, *HDL* high-density lipoprotein.

^a^1st quantile of medical insurance premiums and medical aid beneficiaries.

^b^Exercise > 20 min per time in a week.

### Statin use and the risk for IPF

Among the 10,568 patients in the IPF cohort, 3072 patients (29.1%) were statin users and, among the 31,704 subjects in the control cohort, 8569 subjects (27.0%) were statin users. When adjusted for covariates including comorbidities, statin use was associated with a lower risk for IPF (adjusted OR 0.847, 95% CI 0.800–0.898) than statin nonuse. The protective effects of statin use were consistent regardless of smoking history and sex (Table 2).

**Table 2:** Association between statin use and the risk for IPF.

	IPF	Non-IPF	Unadjusted odds ratio (95% CI)	Adjusted odds ratio (95% CI)
Overall population	N = 10,568	N = 31,704		
Statin nonuser	7496 (70.9%)	23,135 (73.0%)	1.0 (ref)	1.0 (ref)
Statin user	3072 (29.1%)	8569 (27.0%)	1.106 (1.054–1.162)	0.847 (0.800–0.898)
Never smoker	N = 5379	N = 17,855		
Statin nonuser	3814 (70.9%)	12,745 (71.4%)	1.0 (ref)	1.0 (ref)
Statin user	1565 (29.1%)	5110 (28.6%)	1.023 (0.957–1.095)	0.805 (0.745–0.871)
Ever smoker	N = 5189	N = 13,849		
Statin nonuser	3682 (71.0%)	10,390 (75.0%)	1.0 (ref)	1.0 (ref)
Statin user	1507 (29.0%)	3459 (25.0%)	1.229 (1.145–1.320)	0.903 (0.829–0.984)
Male	N = 7269	N = 21,807		
Statin nonuser	5231 (72.0%)	16420 (75.3%)	1.0 (ref)	1.0 (ref)
Statin user	2038 (28.0%)	5387 (24.7%)	1.188 (1.119–1.261)	0.865 (0.806–0.929)
Female	N = 3299	N = 9897		
Statin nonuser	2265 (68.7%)	6715 (67.8%)	1.0 (ref)	1.0 (ref)
Statin user	1034 (31.3%)	3182 (32.2%)	0.964 (0.885–1.049)	0.811 (0.735–0.894)

In the adjusted analysis, multivariable logistic regression was performed by adjusting for the following confounding variables: age, sex, smoking history, drinking habits, body mass index, income, and presence of comorbidities (hypertension, diabetes, ischemic heart disease, chronic kidney disease, and stroke).

### Statin use and clinical outcomes of IPF

Among the 10,568 patients in the IPF cohort, 9784 patients who did not experience myocardial infarction or stroke before the diagnosis of IPF were analyzed to assess the effects of statins on clinical outcomes (Fig. 1). Significant differences were observed in baseline characteristics between statin users and nonusers (Table 3). Statin users were older and cardiovascular comorbidities, such as hypertension and diabetes, were significantly more common among statin users, with almost more than twice the frequency.

**Table 3:** Comparison of statin users and nonusers among patients with IPF.

Variables	Statin user (N = 2571)	Statin nonuser (N = 7213)	*P* value
Age (yr)	68 ± 9	64 ± 13	< 0.001
Male sex	1683 (65.5)	5026 (69.7)	< 0.001
Body mass index (kg/m^2^)	24.6 ± 3.0	23.2 ± 3.2	< 0.001
Smoking status			
Never smoker	1315 (51.2)	3666 (50.8)	< 0.001
Former smoker	761 (29.6)	1781 (24.7)	
Current smoker	495 (19.2)	1766 (24.5)	
Alcohol drinking			
No drinking	1775 (69.1)	4419 (61.2)	< 0.001
Light drinking	476 (18.5)	1579 (21.9)	
Moderate drinking	183 (7.1)	682 (9.5)	
Heavy drinking	137 (5.3)	533 (7.4)	
Low income^a^	572 (22.3)	1636 (22.7)	0.652
Regular exercise^b^	581 (22.6)	1360 (18.9)	< 0.001
Urban residency	1429 (55.6)	4172 (57.8)	0.047
Comorbidity			
Hypertension	1863 (72.5)	2834 (39.3)	< 0.001
Diabetes mellitus	1081 (42.1)	1144 (15.9)	< 0.001
Chronic kidney disease	435 (16.9)	616 (8.5)	< 0.001
Laboratory results			
Total cholesterol	181 ± 47	188 ± 33	< 0.001
LDL cholesterol	102 ± 41	111 ± 30	< 0.001
HDL cholesterol	52 ± 14	52 ± 18	0.088
Fasting glucose	112 ± 34	101 ± 26	< 0.001

Data are presented as mean ± standard deviation and number (%) as indicated.

*LDL* low-density lipoprotein, *HDL* high-density lipoprotein.

^a^1st quantile of medical insurance premiums and medical aid beneficiaries.

^b^Exercise > 20 min per time in a week.

The clinical outcomes of the two groups were compared and analyzed (Table 4). When adjusted for covariates, statin users exhibited lower overall mortality than statin nonusers (adjusted HR 0.779, 95% CI 0.709–0.856). When the time to first hospitalization or ER visit for any cause was analyzed, no significant difference was observed between the two groups (adjusted HR 0.974, 95% CI 0.908–1.044). However, the risk for hospitalization or ER visits due to respiratory causes was lower in statin users than in statin nonusers (adjusted HR 0.818, 95% CI 0.728–0.920).

**Table 4:** Association between statin use and clinical outcomes among patients with IPF.

	Number of patients	Number of events	Person-years	Incidence rate per 1000 person-years	Unadjusted hazard ratio (95% CI)	Adjusted hazard ratio (95% CI)
Overall mortality						
Statin nonuser	7213	1976	28,412	69.5	1.0 (ref)	1.0 (ref)
Statin user	2571	659	9103	72.4	1.007 (0.922–1.100)	0.779 (0.709–0.856)
First hospitalization or visit to emergency room (All causes)						
Statin nonuser	7213	3396	21,317	159.3	1.0 (ref)	1.0 (ref)
Statin user	2571	1279	6536	195.7	1.189 (1.115–1.268)	0.974 (0.908–1.044)
First hospitalization or visit to emergency room (Respiratory causes)						
Statin nonuser	7213	1283	26,587	48.3	1.0 (ref)	1.0 (ref)
Statin user	2571	433	8535	50.7	1.020 (0.914–1.137)	0.818 (0.728–0.920)
Incident myocardial infarction or stroke						
Statin nonuse	7213	464	27,589	16.8	1.0 (ref)	1.0 (ref)
Statin user	2571	203	8771	23.1	1.346 (1.141–1.588)	1.013 (0.848–1.210)

In the adjusted analysis, multivariable Cox proportional hazards regression was performed by adjusting for the following confounding variables: age, sex, smoking history, drinking habits, body mass index, income, and presence of comorbidities (hypertension, diabetes, and chronic kidney disease).

### Impact of duration of statin use

**Figure 2:**
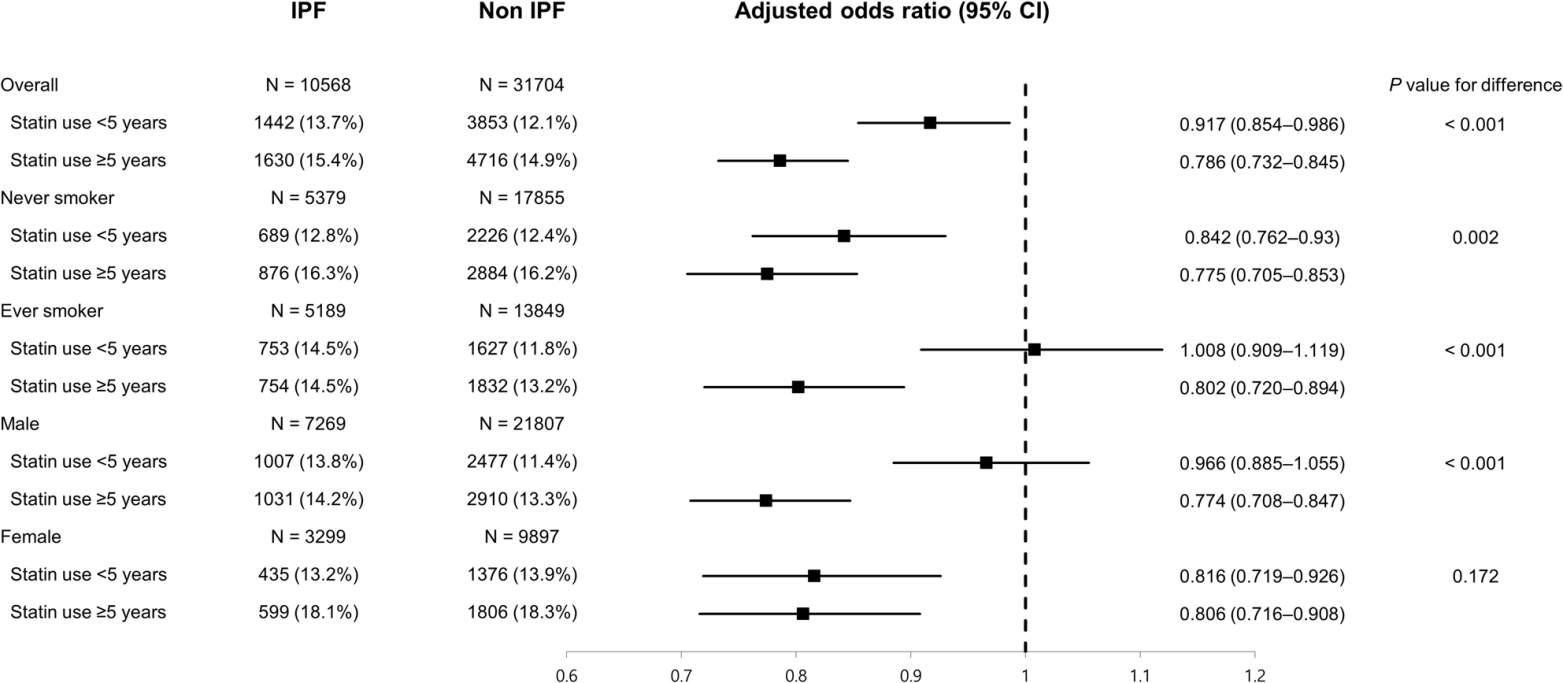
Association between statin use and risk for IPF according to duration of statin use.

**Figure 3:**
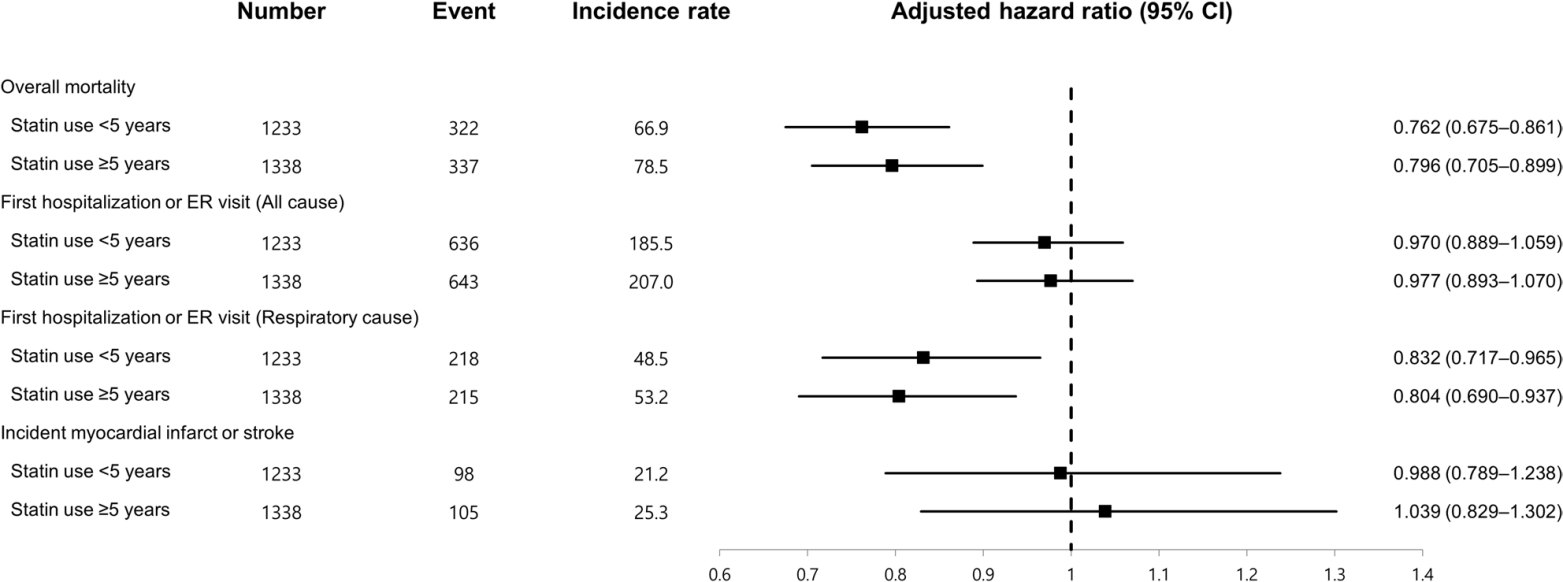
Association between statin use and clinical outcomes of IPF according to duration of statin use.

We evaluated whether the duration of statin use influenced the association between statin use and the risk for and clinical outcomes of IPF (Fig. 2). When statin nonusers were regarded as the reference group, statin use ≥ 5 years was associated with a lower risk for IPF (adjusted OR 0.786, 95% CI 0.732–0.845) than statin use < 5 years (adjusted OR 0.917, 95% CI 0.854–0.986), and the difference was statistically significant (*P* < 0.001). This trend was more pronounced in ever-smokers (*P* < 0.001) and male subjects (*P* < 0.001). In particular, in the subgroup of ever-smokers and male subjects, statin use < 5 years was not associated with a lower risk for IPF than statin nonusers (adjusted OR 1.008, 95% CI 0.909–1.119 and 0.966, 95% CI 0.885–1.055, respectively). However, when an analysis was performed to evaluate the association between statin use and clinical outcomes, the degrees of association were similar regardless of the duration of statin use (Fig. 3).

## Discussion

In this study, we evaluated the association between statin use and the risk for IPF, and the effects of statin use on the clinical outcomes of IPF, using large-scale, nationwide cohort data. When case–control analysis was performed by matching patients with IPF with the control cohort according to age and sex, statin use was associated with a lower risk for IPF than statin nonuse. In addition, in the IPF cohort, statin use was associated with a lower risk for overall mortality and first hospitalization or ER visits related to respiratory causes.

When investigating the causes or risk factors for a specific disease, a longitudinal prospective cohort study involving healthy subjects and monitoring the development of the disease is the best option. However, for rare diseases with low incidence, such as IPF, it is not easy to perform such a study because it would require a very large number of subjects and a follow-up period of several decades, hence a case–control study is a good alternative^14^. As such, we performed a case–control study to investigate whether statin use could alter the risk for IPF and demonstrated promising results, suggesting a protective role of statins against IPF.

Repetitive alveolar microinjuries play a key role in the pathogenesis of IPF and result from multiple genetic and environmental risk factors^15^. While diabetes has been suggested to be associated with IPF^16^, metformin, a representative drug for diabetes, was shown to attenuate pulmonary fibrosis in an *in-vivo* model^17^. However, data regarding whether a specific drug can modify the risk for IPF are scarce. In our case–control analysis, statin use was associated with a lower risk for IPF than statin nonuse. Considering that statins have a favorable safety profile and are widely used in the general population, further studies should be performed to determine whether our findings are reproducible.

Nevertheless, our study has a limitation in that we used a dataset consisting of case and control cohorts matched only for age and sex. There may have been unmeasured biases despite our effort to adjust for confounding factors through multivariable analysis. Although incorporating all available variables for matching would have resulted in more balanced samples, this was practically unattainable because we could not have access to the complete NHIS dataset covering the entire Korean population.

In addition, our study is limited in that we were unable to determine the precise timeframe required for statins to exert a certain pharmacologic effect on the risk of developing IPF. While we attempted to provide a clear definition of statin exposure by classifying patients based on whether statins were prescribed within 1 year before their diagnosis of IPF, we recognize the limitation that statin use for a wide range of durations would have been grouped together as statin users. Exposure to statins over only a few years may not be sufficient to significantly influence the risk of IPF. To address this concern, we performed a subgroup analysis based on the duration of statin use, which revealed that statin use ≥ 5 years conferred a greater protective effect regarding the risk of IPF compared with statin use < 5 years. It is likely that a longer period of time is required for a particular drug to modify the risk of IPF, as supported by a recent study showing that approximately 3 years was required for significant progression of ILA^18^.

Recently, ILA has been increasingly detected and recognized on chest computed tomography among asymptomatic population. ILA is classified into fibrotic and non-fibrotic types, and fibrotic ILA can be a potential precursor to IPF^19^. However, it remains unclear how to prevent disease progression in the subclinical status of ILA, and systematic close follow-up is recommended for high-risk subjects^20^. Currently approved anti-fibrotic drugs, pirfenidone and nintedanib, would not be appropriate in the setting of ILA, given the non-negligible costs and potential drug toxicities. The results of our study suggest that statins may merit investigation of their role in preventing the progression of ILA to IPF.

The protective effects of statins against IPF are biologically plausible in several respects. IPF is known to be a disease of the aging lung, with telomere shortening as one of the contributing factors, which makes the lungs more susceptible to maladaptive responses to alveolar micro-injuries^21^. There is some evidence that statins have anti-aging effects linked to their ability to inhibit telomere shortening^22^. These pleiotropic effects of statins, beyond their lipid-lowering effects, may play a protective role against the development of IPF. Interestingly, in our study, statin use ≥ 5 years was associated with a lower risk for IPF in ever-smokers, whereas statin use < 5 years was not. Given the results of previous studies reporting that cigarette smoking could result in shorter telomeres^23^, longer use of statins may be needed to offset the detrimental effects of cigarette smoking in ever-smokers.

Among patients with newly diagnosed IPF, statin use at baseline was associated with improvement in clinical outcomes in our study compared to statin nonuse, especially for overall survival. There have been a few attempts to evaluate the impact of statin therapy on clinical outcomes in IPF, but a meta-analysis of these studies could not draw a definitive conclusion on the effects of statin use on overall mortality^13^. In fact, most of these previous studies had limited sample sizes, usually including hundreds of patients^8–12^. Our study included approximately 10,000 patients with IPF, which provided greater statistical power. Although statin users had more cardiovascular comorbidities, which was not surprising, when these confounding factors were adjusted for, statin use was associated with lower overall mortality. However, there is a possibility of the presence of unmeasured confounding factors in our analysis. Because our analysis was retrospective and relied on the NHIS database, which is a health claims database, this study cannot completely eliminate every potential bias. To fully address this concern, it would be imperative to perform a propensity score-matched analysis within a meticulously established cohort of patients with IPF or, ideally, to conduct a well-designed prospective study.

There is some experimental evidence supporting the anti-fibrotic potential of statins^24–26^, which are also known to have anti-inflammatory effects^27^. Both anti-fibrotic and anti-inflammatory effects may be beneficial in slowing the progression of IPF. However, in our study, although we demonstrated that overall survival was better in statin users among patients with IPF, it is not clear whether this finding was due to differences in IPF-related mortality or mortality related to other causes, because we could not determine the cause of death in this study. Considering that cardiovascular diseases and lung cancers are also the main causes of death in patients with IPF^28^, the beneficial effects of statins on overall mortality may stem from their impact on these conditions. Statins are well-known to have cardiovascular protective effects and, in addition, a recent study using the Taiwan national health insurance database reported that statin use was associated with lower risk for lung cancers in patients with interstitial lung diseases^29^. However, our finding that statin use was associated with a lower risk for events caused by respiratory causes indicates that statins may have beneficial effects on IPF itself.

The strength of our study is that we used representative nationwide data from a large number of patients captured from real-world clinical practice. However, this study had some limitations that should be addressed. First, although our study suggested a protective role of statins against the risk for IPF, a case–control study could not confirm a causal relationship. The effects of statins on the prognosis of patients with IPF could not be definitively addressed in our study due to its retrospective design. As such, a well-designed, prospective study should be conducted in the future. Second, about 50% of patients who met the operational diagnosis of newly diagnosed IPF was excluded in the final analysis. The most common cause of exclusion was because they did not undergo health screening program within 2 years. It can be suggested that patients with relatively higher health motivation were included in this study. Third, the NHIS database did not have information about the severity of IPF, such as lung function parameters. Therefore, we could not fully adjust for confounding factors when analyzing the clinical outcomes of patients with IPF. Fourth, we could not assess the effects of anti-fibrotic therapy. This is because in Korea, pirfenidone only began to be covered by the National Health Insurance in October 2015, and nintedanib has not yet been covered. Given that our study period was up to December 2017, we did not have sufficient data regarding the use of anti-fibrotic drugs. However, we suspect that the prescription of anti-fibrotic drugs would not have been so different, regardless of whether patients were statin users. Considering the mortality-reducing effects of anti-fibrotic therapy^30^, future studies should gather information about the use of anti-fibrotic drugs when evaluating whether a specific drug could improve the prognosis of IPF. Fifth, our study only investigated the impact of statin use preceding the diagnosis of IPF. Given the possibility that some patients may have started statin therapy after the diagnosis of IPF, statistical methods such as time-varying Cox regression or landmark time analysis may be useful to addressing this issue^31^. Sixth, the NHIS database used for this study predominantly covered only Asian populations, limiting our ability to evaluate patients of diverse ethnic backgrounds. Further studies should be performed in other cohorts from different countries. Seventh, we employed unconditional logistic regression in the analysis of our case–control matched dataset, under the premise that our matching, which was limited to only age and sex, were comparatively loose^32,33^. The absence of conditional matched regression in our statistical methodology raises concerns regarding potential biases in our results. Finally, because this study was based on information gathered from a database, misclassification related to statin use and/or diagnosis of IPF could have occurred. A prescription history of statins may not necessarily mean that the patient actually took the drug. However, in clinical practice, statins are usually taken continuously, unless there are serious side effects, which are relatively rare. Thus, the chances of exposure misclassification appear to be low. In addition, the diagnosis of IPF is strictly reviewed for registration in Korea, because patients with ICD-10 codes for IPF are financially supported by the government. Furthermore, we included only patients diagnosed at referral hospitals, which makes us more confident in the reliability of IPF diagnosis. However, relying solely on the health claims database and utilizing ICD codes for identifying cases with IPF presents inherent limitations, primarily due to our limited ability to verify the diagnostic accuracy.

In conclusion, this large, nationwide, population-based cohort study conducted in a real-world setting found that statin use was associated with a lower risk for IPF than statin nonuse. In addition, statin use was associated with improved overall survival and reduced risk for respiratory-related hospitalization or ER visits among patients with IPF. Prospective studies aiming to confirm the potential beneficial effects of statins on IPF are warranted.

## Methods

### Study data source

This study used data from the health claims database established by the NHIS of South Korea, a single insurer managed by the Korean government. Because it is mandatory for every Korean citizen to subscribe to the NHIS, the NHIS database contains extensive health-related data from the entire Korean population, including personal and sociodemographic information, data regarding every inpatient and outpatient service, prescriptions, and mortality data^34^.

In South Korea, the NHIS has been providing national health screening checkup programs since 1995 to improve the health status of the general population. Health-related data obtained through this health screening program are also available in the NHIS database^35^. The data and materials of the NHIS are accessible to the public and are widely used by medical researchers. Requirement for informed consent was waived by the institutional review board of Seoul National University Hospital because information regarding personal identification was completely removed while establishing the database. The study protocol was approved by the institutional review board of Seoul National University Hospital (IRB No. E-1904-001-1020), and all methods were carried out in accordance with relevant guidelines and regulations.

### Study design and population

The present study was a retrospective analysis based on data from the NHIS database, which included two distinct parts. The first is the case–control study comparing patients with IPF and control populations, and the second is the retrospective cohort study focusing only on the IPF cohort to evaluate the beneficial effect of statins in IPF.

A case–control study was performed to evaluate the association between statin use and risk for IPF. For this purpose, a cohort of patients with newly diagnosed IPF was established. First, patients who had medical claims with International Classification of Diseases (ICD-10) codes for IPF (J841 or J8418) between January 2010 and December 2017 were screened. We chose to exclude patients diagnosed prior to 2010 given the considerable differences in treatment strategies and clinical outcomes between patients diagnosed in the earlier period and those diagnosed more recently^36^. Subsequently, an operational definition of newly diagnosed IPF, as detailed in our previous study, was applied^37^.

Briefly, we first searched for patients in whom ICD-10 codes for IPF were registered by physicians working in referral hospitals, not primary care clinics, considering that an accurate diagnosis of IPF requires multidisciplinary discussion. Among these, patients with claims for chest computed tomography (CT) and pulmonary function tests within 1 year and 6 months, respectively, before the first registration of ICD-10 codes for IPF were included. Patients with ICD-10 codes for autoimmune or connective tissue diseases, or other pulmonary diseases were excluded. Among the selected patients fulfilling this operational definition, only those who had participated in national health screening programs within 2 years before the diagnosis of IPF were considered to be eligible for this study to use the health-related information that could be obtained through the programs, such as smoking history.

Study patients were classified into statin users and nonusers according to whether they were being prescribed statins at the time of IPF diagnosis. An operational definition of statin use was applied for this purpose because the exact timeframe for statins to exert potential effects on the risk of IPF is not certain. A patient was regarded to be a statin user if statins were prescribed at least twice within 1 year before the index date, the date of the first registration of the ICD-10 codes for IPF. To define only those who had never been exposed to statins as statin nonusers, patients in whom statins were prescribed only once within 1 year before the index date or statins were ever prescribed before but not within 1 year before the index date were excluded.

Following the establishment of the IPF cohort, another control cohort without IPF, 1:3 matched according to age and sex, was established using the exact matching algorithm. The control population was selected without replication among subjects without ICD-10 codes for IPF. Statin users and nonusers were defined as those in the IPF cohort. The index date for the control population was determined as the index date for the matched case patients.

After conducting a case–control study, a subsequent analysis was performed using follow-up data from study patients in the IPF cohort as a retrospective cohort study. We evaluated the association between statin use and clinical outcomes. For this analysis, patients who had already been diagnosed with myocardial infarction or stroke before the index date were excluded because incident myocardial infarction or stroke was one of the outcome events of interest.

### Study outcome and covariates

In the case–control study, the association between statin use and the risk for IPF was assessed by calculating odds ratios (ORs). After matching for age and sex, additional adjustments were performed for the following covariates: smoking history, alcohol consumption pattern, body mass index, income level, and comorbidities including diabetes, hypertension, ischemic heart disease, stroke, and chronic kidney disease. The presence of comorbidities was assessed using the relevant ICD-10 codes for each disease. Covariates were selected among clinically relevant variables that could be associated with statin use and identified through the NHIS database.

Using only the IPF cohort, we investigated whether statin use was associated with improvement in clinical outcomes, including overall mortality, hospitalization or emergency room (ER) visits, and incident myocardial infarction or stroke. After the overall events of hospitalization or ER visits were assessed, events associated with a primary diagnosis of respiratory diseases were further analyzed using ICD-10 codes of J00–J99. However, the cause of mortality could not be identified through our database. The covariates adjusted for were similar to those used in the case–control analysis and included smoking history, alcohol consumption pattern, body mass index, income level, and comorbidities including diabetes, hypertension, and chronic kidney disease.

### Statistical analysis

Descriptive statistics were used to summarize the baseline characteristics of the study population. Continuous variables were summarized as means with standard deviations and categorical variables were reported as frequencies (percentages). The association between statin use and the risk for IPF was evaluated using multivariable logistic regression, and adjusted ORs were reported with corresponding 95% confidence intervals (CIs). Subgroup analysis according to smoking history and sex was performed as well.

The association between statin use and clinical outcomes among patients in the IPF cohort was evaluated using Cox proportional hazard regression, and adjusted hazard ratios (HRs) were calculated with corresponding 95% CIs. If a patient started statin therapy before development of the events of interest, further follow-up was censored.

Given that the operational definition of statin use could not provide detailed information about the duration of drug use, additional analysis was performed by dividing statin users according to the duration of drug use, using 5 years as the cut-off point (statin use ≥ 5 years vs. < 5 years vs. nonuse). We compared the effects of statin use ≥ 5 years and statin use < 5 years by including an interaction term in the regression model. SAS version 9.4 (SAS Institute, Cary, NC, USA) was used for statistical analyses, and *P* values < 0.05 for two-tailed tests were considered to be statistically significant.

## Data Availability

The data that support the findings of this study are available from NHIS database, but there are restrictions applied to the availability of these data, which were used under license for the current study and so are not currently publicly available. Data are however available from the corresponding author upon reasonable request and with permission of NHIS.

## Abbreviations

HMG-CoA: 3-hydroxy-3-methylglutaryl coenzyme A
CI: Confidence interval
ER: Emergency room
HR: Hazard ratio
ICD: International classification of diseases
ILA: Interstitial lung abnormality
IPF: Idiopathic pulmonary fibrosis
NHIS: National health insurance service
OR: Odds ratio
